# Surgical or non-surgical treatment of traumatic skeletal fractures in adults: systematic review and meta-analysis of benefits and harms

**DOI:** 10.1186/s13643-020-01424-4

**Published:** 2020-08-13

**Authors:** Søren T. Skou, Carsten B. Juhl, Kristoffer B. Hare, L. Stefan Lohmander, Ewa M. Roos

**Affiliations:** 1 Research Unit for Musculoskeletal Function and Physiotherapy, Department of Sports Science and Clinical Biomechanics, University of Southern Denmark, Odense, Denmark; 2 Department of Physiotherapy and Occupational Therapy, Næstved-Slagelse-Ringsted Hospitals, Region Zealand, Slagelse, Denmark; 3 Department of Rehabilitation, Copenhagen University Hospital, Herlev and Gentofte, Denmark; 4 Department of Orthopedics, Næstved-Slagelse-Ringsted Hospitals, Region Zealand, Slagelse, Denmark; 5 Department of Regional Health Research, University of Southern Denmark, Odense, Denmark; 6 Department of Clinical Sciences Lund, Orthopedics, Lund University, Lund, Sweden

**Keywords:** Systematic review, Fracture, Therapeutics, Orthopedics, Randomized, controlled trial

## Abstract

**Background:**

A comprehensive overview of treatments of common fractures is missing, although it would be important for shared decision-making in clinical practice. The aim was to determine benefits and harms of surgical compared to non-surgical treatments for traumatic skeletal fractures.

**Methods:**

We searched Medline, Embase, CINAHL, Web of Science, and CENTRAL until November 2018, for randomized trials of surgical treatment in comparison with or in addition to non-surgical treatment of fractures in adults. For harms, only trials with patient enrollment in 2000 or later were included, while no time restriction was applied to benefits. Two reviewers independently assessed studies for inclusion, extracted data from full-text trials, and performed risk of bias assessment. Outcomes were self-reported pain, function, and quality of life, and serious adverse events (SAEs). Random effects model (Hedges’ g) was used.

**Results:**

Out of 28375 records screened, we included 61 trials and performed meta-analysis on 12 fracture types in 11 sites: calcaneus, clavicula, femur, humerus, malleolus, metacarpus, metatarsus, radius, rib, scaphoideum, and thoraco-lumbar spine. Seven other fracture types only had one trial available. For distal radius fractures, the standardized mean difference (SMD) was 0.31 (95% CI 0.10 to 0.53, *n* = 378 participants) for function, favoring surgery, however, with greater risk of SAEs (RR = 3.10 (1.42 to 6.77), *n* = 436). For displaced intra-articular calcaneus fractures, SMD was 0.64 (0.13 to 1.16) for function (*n* = 244) and 0.19 (0.01 to 0.36) for quality of life (*n* = 506) favoring surgery. Surgery was associated with a smaller risk of SAE than non-surgical treatment for displaced midshaft clavicular fractures (RR = 0.62 (0.42 to 0.92), *n* = 1394). None of the other comparisons showed statistical significance differences and insufficient data existed for most of the common fracture types.

**Conclusions:**

Of 12 fracture types with more than one trial, only two demonstrated a difference in favor of surgery (distal radius fractures and displaced intra-articular calcaneus fractures), one of which demonstrated a greater risk of harms in the surgical group (distal radius fractures). Our results highlight the current paucity of high-quality randomized trials for common fracture types and a considerable heterogeneity and risk of bias in several of the available trials.

**Systematic review registration:**

PROSPERO CRD42015020805

## Background

Fractures are an important public health burden. The age-standardized annual fracture incidence in England has been reported to be as high as 3.6% [1] with great variation dependent on how and from which population it is estimated [1–3]. Years lived with disability due to fractures are estimated to be around 22 million, most of which are long-term disability [4], and the total UK annual hospital costs associated with incident hip fractures in older adults alone are around £1.1 billion [5].

Surgery is the preferred treatment of most displaced fractures, but evidence from recent years suggests that non-surgical treatment might serve as an effective alternative for selected fractures, potentially associated with fewer adverse events and lower costs [6–9].

Fractures of the clavicula, humerus, radius, ulna, metacarpals, femur, and ankle are some of the most common fractures [2, 3]. However, a comprehensive overview of the benefits (e.g., improvements in pain, function, and quality of life) and harms (e.g., serious adverse events) of surgical and non-surgical treatment of these and other fractures is missing. A better understanding of the benefits and harms of these treatments for each of the most common fractures separately would serve as an important basis for shared decision-making about treatment of fractures in clinical practice.

We therefore aimed in this systematic review and meta-analysis to determine the benefits and harms of surgical compared with non-surgical treatments for acute, traumatic, skeletal fractures in adults. We extend existing knowledge [6–9] by including more recent trials and by including and analyzing outcomes on patient-reported pain, physical function, quality of life, and SAE on each type of fracture separately.

## Methods

This report conforms to the PRISMA statement [10]. The study followed the published guidelines on systematic reviews from the Cochrane Collaboration [11] and it was pre-registered with PROSPERO (CRD42015020805). In the PROSPERO-registration, two systematic reviews are described, the other being a systematic review of surgical vs. non-surgical treatment of non-fracture musculoskeletal conditions, which will be reported in a subsequent publication.

### Search strategy

Two authors (STS + CBT) searched MEDLINE via PubMed, EMBASE via Ovid, CINAHL (including preCINAHL) via EBSCO, Web of Science via Web of Knowledge and CENTRAL, all up to 5 November 2018. We included trials reported in English, German, Danish, Swedish, and Norwegian (i.e., languages that the authors understand). For SAEs, only trials enrolling patients from 2000 were included due to the increasing quality of surgery and anesthesia and with the expectation of improved reporting of SAEs following the CONSORT statement published in 1996 and updated in 2001. No time restriction was applied for benefits. The search strategies were adjusted according to the specifications of the individual database (see Additional file S1). Reference lists of included articles and the most recent systematic reviews were reviewed to identify additional trials.

### Trial selection

Two authors (STS + CBJ) independently assessed titles/abstracts for trial eligibility using a priori selection criteria. The full text was retrieved if found eligible by at least one reviewer. The same authors independently evaluated eligibility of the retrieved full-text trials. Consensus was reached by discussion.

We included randomized trials conducted in any setting evaluating the effect of surgical treatment in comparison or in addition to non-surgical treatment of traumatic fractures in adults (mean age of trial participants 18+) with data on patient-reported pain, physical function, quality of life or SAEs. If any of these outcomes were reported, with data available that could be used in a meta-analysis, the trial was included. Surgery was pre-defined as any procedure that both changes the anatomy and requires a skin incision or use of an endoscopic technique [12], while non-surgical treatment was defined as all non-surgical treatments and placebo treatments.

Trials investigating the effects of drug substances used perioperatively, vertebroplasty, and kyphoplasty, cancer-related fractures, and jaw fractures were excluded. Conference abstracts were also excluded.

### Outcomes

Our pre-defined outcomes of interest for benefit were patient-reported pain, physical function, and quality of life, and SAEs for harm. If more than one outcome was available for patient-reported pain, physical function, and quality of life, multidimensional outcomes were preferred before unidimensional outcomes. For unidimensional pain, pain intensity in the activity was preferred over pain intensity in rest. We pre-defined SAEs using the U.S. Food and Drug Administration definition, as all adverse events having the potential to significantly compromise the clinical outcome, result in significant disability or incapacity, requiring inpatient or outpatient hospital care, and those considered to prolong hospital care, to be life-threatening, or to result in death [13]. Non-unions were considered as SAE, while mal-unions were only considered as SAE if this resulted in additional treatment or significant disability or pain. Minor additional surgery such as removal of Kirschner wires was not considered an SAE, if they were part of normal clinical practice following the specific surgical procedure. Crossovers from non-surgical to surgical treatment were not considered an SAE unless caused by an SAE.

### Data extraction

A customized data extraction form was developed for the outcomes, and two authors (STS + CBJ) independently extracted data. We preferred data from the 12 months follow-up of the trials, as this is a very common primary endpoint in trials of orthopedic surgery and as benefits from surgical and non-surgical treatment are expected to be stable at that time point. If data was not available from a 12-month follow-up, data from the follow-up closest to 12 months was used. We extracted the number of patients randomized to each treatment, age, sex, study location (country), pain, and BMI at baseline, fracture type, surgical and non-surgical intervention, follow-up time, number of patients not undergoing surgery in the surgical group, number of crossover to surgical treatment, number of patients analyzed, mean effect and SD, deaths and SAEs during follow-up and types of SAEs. If SAEs, deaths, or crossover were not mentioned, it was considered as if it had not occurred.

### Risk of bias assessment

Risk of bias was assessed using the Risk of Bias 2.0 tool from the Cochrane Collaboration on trials with results on benefits [14]. Two authors (STS + CBJ) independently assessed if each of the following five domains was associated with low risk of bias, some concerns or high risk of bias: (1) bias arising from the randomization process, (2) bias due to deviations from intended interventions, (3) bias due to missing outcome data, (4) bias in measurement of the outcome, (5) bias in selection of the reported result. If four or five of the individual domains were found to be associated with some concerns of risk of bias, or if one of them was associated with a high risk of bias, the overall risk of bias was rated as high risk.

For SAEs (including death) trial quality was assessed independently on trials with results on SAEs by two authors (STS + CBJ) using the 15-point McMaster tool for assessing quality of harms assessment and reporting in study reports (McHarm) [15]. A score greater than 9 was considered a high score and indicative of low risk of bias.

Any discrepancies in the assessment of trial quality were resolved by discussion.

### Data synthesis and statistical methods

The benefits of surgery were estimated using meta-analyses as the standardized mean difference (SMD) allowing for pooling the various outcomes assessed in the individual trials. The SMD was estimated as the difference in mean at follow-up in the intervention and control groups divided by the pooled SD. If the SD was not available it was estimated from the standard error, confidence interval, or the *P* value, as recommended in the Cochrane Handbook [11]. If necessary, means and measures of dispersion were estimated from figures in the included trials. If only SD of the baseline score and SD of the change score were available, these were used for estimating SD of the final score [11]. SMD was adjusted to Hedges’ g, as Cohen’s d overestimate the effect in small studies. The SMD was interpreted clinically as originally proposed by Cohen [16], i.e., a SMD of 0.2 was small, a SMD of 0.5 was moderate, and a SMD of 0.8 was large. Heterogeneity was estimated as between-study variance (tau^2^) and *I*-squared measuring the proportion of variation (i.e., inconsistency) in the combined estimates due to between-study variance. When *I*-squared is 0%, no inconsistency is seen between results of individual trials and inconsistency is maximal when *I*-squared is 100%.

SAEs were calculated as relative risk (RR). In order to handle null findings in either intervention or control group, Battaglias code was imputed. Battaglias code imputes one event distributed according to the numbers in the intervention and control group. The analyses of deaths followed the same approach. Results of individual studies were summed using a random-effects model meta-analysis for studies with relevant data on any of the outcomes, separated based on fracture type, body site, and outcome. While at least two studies were required to conduct meta-analyses on the different fracture types, all studies adhering to the eligibility criteria were included in the systematic review.

A *p* value less than 0.05 (two-sided) was considered significant. Analyses were carried out in Stata 15 (StataCorp, College Station, TX, USA).

## Results

### Description of included trials

**Fig. 1:**
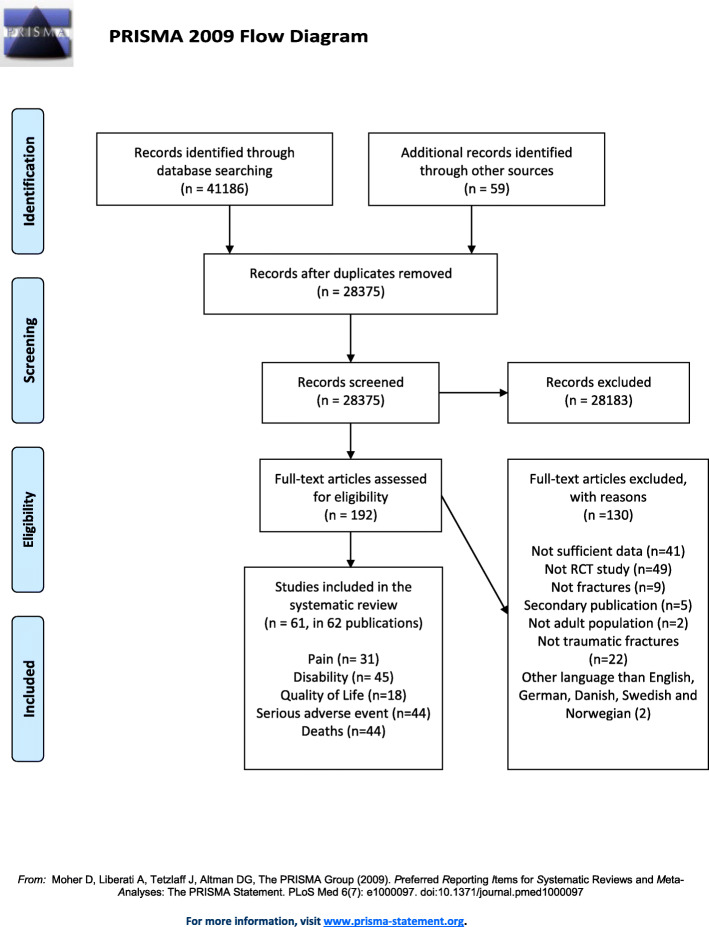
Selection of trials of surgical and non-surgical treatment of fractures

The literature search revealed 41,186 hits and 59 were identified from other sources (i.e., references in systematic reviews and in included studies). After removing duplicates, we screened 28,375 titles and abstracts, which led to the retrieval of 192 full texts. After screening full texts, we ended up with 61 trials (in 62 publications) with relevant data available on either patient-reported pain, function, quality of life, and/or SAEs (Fig. 1). These trials were spread across 19 fracture types at 13 body sites: calcaneal (displaced intra-articular), clavicula (displaced midshaft, other), femur (Pipkin type), humerus (proximal, shaft), malleolar (trimalleolar, unstable (uni- bi- or trimalleolar), stable lateral malleolar, other), metacarpal (5th), metatarsal (5th), radius (distal), rib (flail chest), scaphoid (waist), tibia (shaft), thoraco-lumbar spine (traumatic), ulnar (olecranon and shaft) fractures.

Out of the 61 eligible trials (*n* = 6021 patients), 31 had data on pain (*n* = 2605), 45 on function (*n* = 3735), 18 on quality of life (*n* = 2306), 44 on SAEs (*n* = 3953), and 44 on deaths (*n* = 4145). Displaced midshaft clavicula (*n* = 14 trials), distal radius (*n* = 7), displaced intra-articular calcaneus (*n* = 6), scaphoid waist (*n* = 6), and proximal humerus (*n* = 6) fractures were the fractures most commonly investigated. Trials were carried out across 24 different countries, with the UK (*n* = 11), Sweden (*n* = 9), and the USA (*n* = 6) being the most common. Age and gender distribution varied depending on the fracture type. Table 1 presents the characteristics of the included trials.

**Table 1:** Summary of included trials of surgical and non-surgical treatment of fractures

Fracture type	Author, year, country	Age, % female	Surgical treatment (*n*)	Did not undergo surgery after randomization (*n*)	Non-surgical treatment (*n*)	Received surgical treatment in control group (*n*)	Benefit/harm outcomes	Follow-up time (month)
Calcaneal, displaced intraarticular	Agren, 2013, Sweden [17]	48.5 years, 28.0%	42	0	40	0	Pain, disability, QoL	12 months
	Buckley, 2002 [18] and O’Brien, Canada [19]	40.0 years, 10.3%	206	0	218	0	Pain, QoL	24 months
	Griffin, 2014, UK [20]	46.4 years, 15.9%	68	5	75	3	Disability, QoL SAE	24 months
	Ibrahim, 2007, UK [21]	48.5 years, 19.2%	25	0	31	0	Pain, disability	180 months
	Nouraei, 2011, Iran [22]	49 years,	36	0	36	0	Pain	6 months
	Thordarson, 1996, USA [23]	35.4 years, 19.2%	16	0	14	0	Disability	16 months
Clavicular, displaced midshaft	Ahrens, 2017, UK [24]	36.2 years, 13.6%	143	11	131	16	Disability, SAE	9 months
	Chen, 2011c, China [25]	37.7 years, 46.7%	30	0	30	0	Disability, SAE	15 months
	Judd, 2009, USA [26]	26.5 years, 87.7%	29	0	28	0	Disability, SAE	12 months
	Koch, 2008, Germany [27]	35.4 years, 33.8%	35	0	33	0	Pain, SAE	1 month
	Kumar, 2018, India [28]	N/A	40	0	40	0	SAE	12 months
	McKee, 2007, Canada [29]	33.5 years, 21.6%	66	1	64	1	Disability, SAE	12 months
	Melean, 2015, Chile [30]	37.6 years,	34	0	38	4	SAE	4 months
	Mirzatolooei, 2011, Iran [31]	35.7 years, 18.0%	29	3	31	0	Disability, SAE	12 months
	Qvist, 2018, Denmark [32]	39.5 years, 18.5%	74	1	66	9	Disability, SAE	12 months
	Robinson, 2013, UK [33]	32.4 years, 12.5%	95	0	92	13	Disability, QoL, SAE	12 months
	Smekal, 2009, Austria [34]	37.7 years, 13.3%	33	0	32	3	Disability, SAE	6 months
	Tamaoki, 2017, Brazil [35]	32.5 years, 14.5%	59	0	56	2	Pain, disability, SAE	12 months
	Virtanen, 2012, Finland [36]	36.7 years, 13.3%	28	0	31	1	Pain, disability, SAE	12 months
	Woltz, 2017, Netherlands [37]	37.8 years, 8.8%	86	0	62	12	Disability, QoL, SAE	12 months
Clavicular, other	Dugar, 2013, India [38]	N/A	15	0	15	0	SAE	12 months
	Yadav, 2015, India [39]	33.1 years, 20.0%	13	0	12	0	SAE	3 months
Femoral, caput	Chen, 2011a, China [40]	37.5 years, 18.8%	8	0	8	0	SAE	38 months
	Chen, 2011b, China [41]	38.7 years, 29.2%	12	0	10	2	SAE	39 months
Humeral shaft	Matsunaga, 2017, Brazil [42]	38.7 years, 33.6%	52	0	10	48	Pain, disability, QoL, SAE	12 months
Humeral, proximal	Boons, 2012, Netherlands [43]	78.2 years, 94.0%	25	0	25	0	Pain, disability, SAE	12 months
	Fjalestad, 2014, Norway [44]	72.6 years, 88.0%	25	0	24	1	Disability, QoL SAE	12 months
	Olerud, 2011a, Sweden [45]	73.9 years, 81.4%	27	0	27	1	Pain, Disability, QoL, SAE	12 months
	Olerud, 2011b, Sweden [46]	76.7 years, 85.5%	30	0	30	0	Pain, disability, QoL, SAE	12 months
	Rangan, 2015, UK [47]	66.0 years, 76.8%	109	16	112	13	Disability, QoL, SAE	12 months
	Zyto, 1997, Sweden [48]	74.0 years, 87.5%	20	0	20	5	Pain, disability	50 months
Malleolar, other	Makwana, 2001, UK [49]	66.9 years, 72.1%	22	0	14	8	Pain, disability	27 months
	Willet, 2016, UK [50]	70.6 years, 74.2%	302	7	277	34	Pain, disability, QoL, SAE	6 months
Malleolar, stable	Mittal, 2017, Australia and New Zealand [51]	39.0 years, 51.9%	72	8	78	2	Disability, QoL, SAE	12 months
Malleolar, trimalleolar	Salai, 2000, Israel [52]	78.3 years, 75.0%	46	0	8	30	Pain, disability	38 months
Malleolar, unstable	Sanders, 2012, Canada [53]	41.0 years, 49.4%	41	0	39	1	Disability, QoL, SAE	12 months
Metacarpal, 5th metacarpal neck	Sletten, 2015, Norway [54 ]	27.0 years, 82.3%	38	4	43	0	Disability, SAE	12 months
	Strub, 2010, Switzerland [55]	30.0 years, 5.0%	20	0	20	0	SAE	12 months
Metatarsal, 5th metatarsal neck	Lee, 2016, South Korea [56]	41.7 years, 55.2%	9	0	9	0	Pain	2 months
	Wu, 2018, China [57]	27.1 years, 36.6%	23	0	22	1	Pain, SAE	12 months
Radial, distal	Abbaszadegan, 1990, Sweden [58]	63.0 years, 76.6%	23	0	24	0	Pain, disability	12 months
	Arora, 2011, Austria [59]	76.7 years, 75.3%	45	0	45	0	Pain, disability, SAE	12 months
	Azzopardi, 2005, UK [60]	71.5 years, 88.9%	30	0	27	0	Pain, disability, QoL, SAE	12 months
	Földhazy, 2010, Sweden [61]	71.6 years, 89.8%	28	0	31	0	Pain, disability, SAE	12 months
	Kreder, 2006, Canada and USA [62]	52.9 years, 65.5%	54	0	54	5	Pain, disability	12 months
	Mardani Kivi, 2011, Iran [63]	50.8 years, 13.0%	99	0	93	6	SAE	3 months
	McQueen, 2008, UK [64]	29.4 years, 16.7%	30	0	30	0	SAE	12 months
	Wong, 2010, Hong Kong [65]	70.5 years, 81.7%	31	0	31	0	Pain, disability, QoL, SAE	12 months
Rib, flail chest	Marasco, 2013, Australia [66]	58.5 years, 13.0%	22	1	23	0	Pain, disability, QoL, SAE	6 months
Scaphoid, waist	Arora, 2007, Austria [67]	33.0 years, 27.3%	23	0	24	0	Pain, disability, SAE	6 months
	Clementson, 2015, Sweden [68]	31.4 years, 18.4%	13	1	24	0	disability, SAE	12 months
	Dias, 2005, UK [69]	29.5 years, 10.2%	44	0	37	7	Pain	12 months
	Vinnars, 2008, Sweden [70]	30.5 years, 22.7%	40	3	41	1	Disability	120 months
Thoraco-lumbal, traumatic compression	Piazzolla, 2011, Italy [71]	39.9 years, 36.0%	24	0	26	0	Pain, disability, SAE	12 months
	Shen, 2001, Taiwan [72]	43.2 years, 48.8%	33	7	43	0	Pain, disability	12 months
	Siebenga, 2006, Netherlands [73]	41.8 years, 37.5%	18	0	16	0	Pain, disability	52 months
	Wood, 2003, USA [74]	41.4 years, 31.9%	26	0	26	1	Pain, disability, QoL	46 months
Tibial shaft	Karladani, 2000, Sweden [75]	39.0 years, 32.1%	27	0	12	17	Pain, Disability, QoL	12 months
	Granetzny, 2005, Egypt [76]	38.2 years, 22.5%	20	0	20	0	SAE	2 months
Ulnar shaft	Hussain, 2018, India [77]	38.9 years, 13.3%	20	0	17	3	Disability, SAE	12 months
Ulnar, olecranon	Duckworth, 2017, UK [78]	82.9 years, 89.5%	11	0	1	7	Disability, SAE	12 months

*QoL* quality of life; *SAE* serious adverse events

**Fig. 2:**
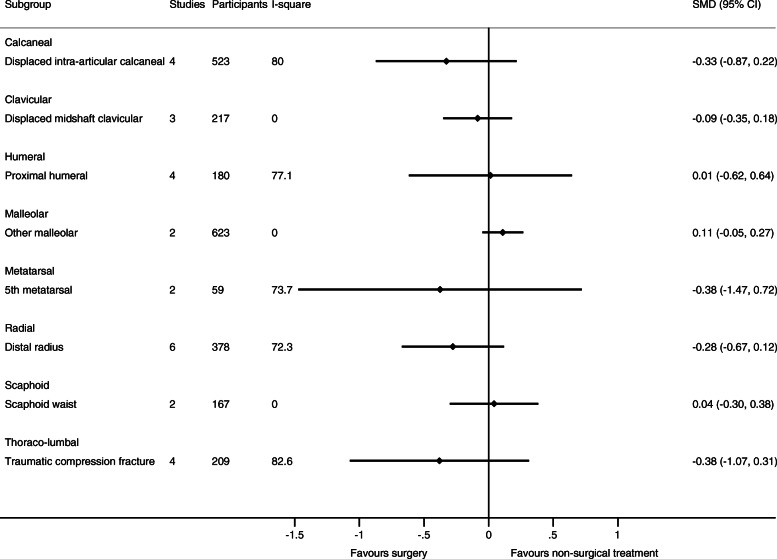
Results of the analysis of effects of surgical and non-surgical treatment on pain. Fracture sites are in alphabetic order

**Fig. 3:**
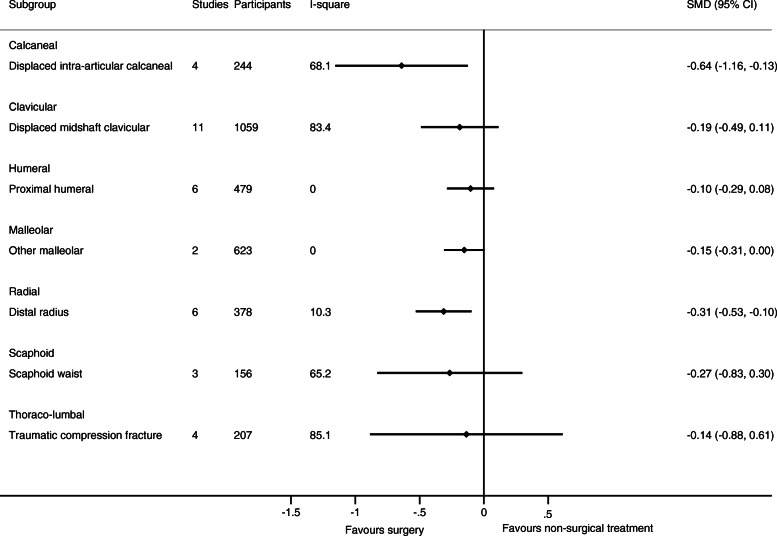
Results of the analysis of effects of surgical and non-surgical treatment on function. Fracture sites are in alphabetic order

**Fig. 4:**
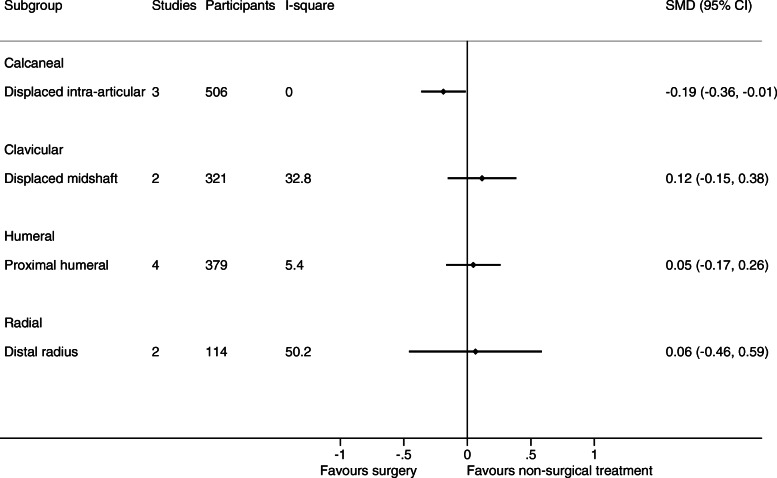
Results of the analysis of effects of surgical and non-surgical treatment on quality of life. Fracture sites are in alphabetic order

**Fig. 5:**
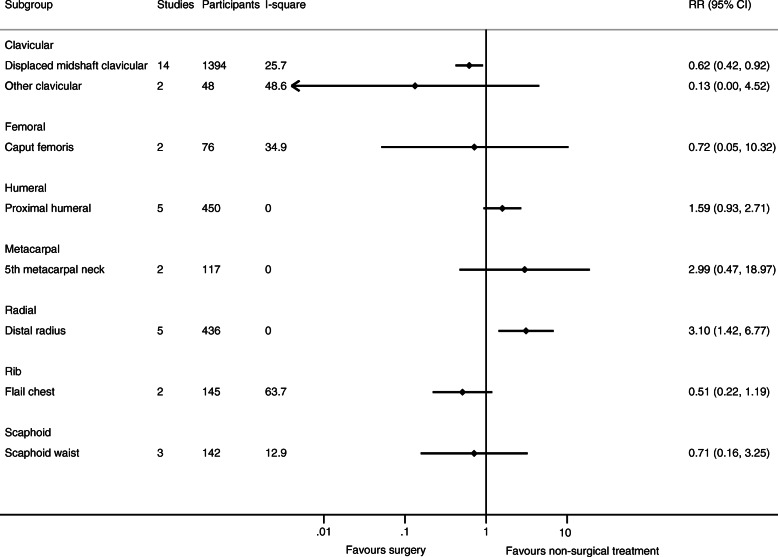
Results of the analysis of effects of surgical and non-surgical treatment on serious adverse events. Fracture sites are in alphabetic order

As only one trial with relevant data was available for humeral shaft, malleolar (trimalleolar, unstable (uni- bi- or trimalleolar), stable lateral malleolar), tibia (shaft), and ulnar (olecranon and shaft) fractures, respectively, only 12 fracture types in 11 body sites were evaluated in meta-analyses. See Figs. 2, 3, 4, and 5 for the number of trials and patients included in the meta-analyses within each of the fracture types for each of the outcomes.

### Benefits

#### Synthesis of results

The results of the meta-analytic syntheses for each of the fracture types separately are presented in Fig. 2 (pain), Fig. 3 (function), and in Fig. 4 (quality of life).

For 6 out of the 8 fracture types with available data on pain, function, and quality of life from at least two trials, no important differences in pain and function were demonstrated between surgical and non-surgical treatment. No studies included a placebo treatment.

For 2 fracture types, surgical treatment was associated with greater benefits. For distal radius fractures (6 trial s[58–62, 65] (*n* = 378)), the SMD was 0.31 (0.10 to 0.53) for function. For displaced intra-articular calcaneus fractures (4 [17, 20, 21, 23] /3 [17, 18, 20] trials (*n* = 244/506), SMD was 0.64 (0.13 to 1.16) for function, and 0.19 (0.01 to 0.36) for quality of life.

Additional file S2 presents the full forest plots for all comparisons.

One trial on trimalleolar ankle fractures (*n* = 65) [52] and one trial on tibial shaft fractures (*n* = 53) [75] also demonstrated a significant effect for function in favor of surgery.

#### Risk of bias

Table 2 presents the risk of bias assessment for the individual trials.

**Table 2:** Assessment of risk of bias of included trials of surgical and non-surgical treatment of fractures

Author, year	Randomization process	Deviations from intended interventions	Missing outcome data	Measurement of the outcome	Selection of the reported result	Overall bias
Abbaszadegan, 1990	Some concern	Some concern	Low risk	Some concern	Some concern	High risk
Agren, 2013	Low risk	Low risk	Low risk	Some concern	Some concern	Some concern
Ahrens, 2017	Low risk	Some concern	Low risk	Some concern	Some concern	Some concern
Arora, 2007	Some concern	Low risk	Some concern	Some concern	Some concern	High risk
Arora, 2011	Low risk	Low risk	Low risk	Some concern	Some concern	Some concern
Azzopardi, 2005	Some concern	Low risk	Some concern	Some concern	Some concern	High risk
Boons, 2012	Low risk	Low risk	Low risk	Some concern	Some concern	Some concern
Buckley, 2002	Low risk	Low risk	High risk	Some concern	Some concern	High risk
Chen, 2011c	Some concern	Low risk	Low risk	Some concern	Some concern	Some concern
Clementson, 2015	Low risk	Some concern	High risk	Some concern	Some concern	High risk
Dias, 2005	Low risk	Low risk	Some concern	Some concern	Some concern	Some concern
Duckworth, 2017	Low risk	Low risk	Some concern	Some concern	Some concern	Some concern
Fjalestad, 2014	Low risk	Low risk	Low risk	Some concern	Some concern	Some concern
Földhazy, 2010	Low risk	Some concern	Some concern	Some concern	Some concern	High risk
Griffin, 2014	Low risk	Low risk	Low risk	Some concern	Low risk	Some concern
Hussain, 2017	Some concern	Some concern	Some concern	Some concern	Some concern	High risk
Ibrahim, 2007	High risk	Some concern	High risk	Some concern	Some concern	High risk
Judd, 2009	Low risk	Low risk	Some concern	Some concern	Some concern	Some concern
Karladani, 2000	Some concern	High risk	Some concern	Some concern	Some concern	High risk
Koch, 2008	Some concern	Some concern	Some concern	Some concern	Some concern	High risk
Kreder, 2006	Low risk	Low risk	Some concern	Some concern	Some concern	Some concern
Kumar, 2018	High risk	Some concern	Some concern	Some concern	Some concern	High risk
Lee, 2016	Some concern	Some concern	Low risk	Some concern	Some concern	High risk
Makwana, 2001	Low risk	Some concern	Some concern	Some concern	Some concern	High risk
Marasco, 2013	Low risk	Low risk	Low risk	Some concern	Some concern	Some concern
Matsunaga, 2017	Low risk	Some concern	Some concern	Some concern	Low risk	Some concern
McKee, 2007	Low risk	Low risk	Some concern	Some concern	Some concern	Some concern
Mirzatolooei, 2011	Low risk	Some concern	Some concern	Some concern	Some concern	High risk
Mittal, 2017	Low risk	Low risk	Some concern	Some concern	Low risk	Some concern
Nouraei, 2011	Some concern	Low risk	Some concern	Some concern	Some concern	High risk
Olerud, 2011a	Low risk	Low risk	Low risk	Some concern	Some concern	Some concern
Olerud, 2011b	Low risk	Low risk	Low risk	Some concern	Some concern	Some concern
Piazzolla, 2011	Some concern	Low risk	Low risk	Some concern	Some concern	Some concern
Qvist, 2018	Low risk	Low risk	Some concern	Some concern	Low risk	Some concern
Rangan, 2015	Low risk	Some concern	Low risk	Some concern	Low risk	Some concern
Robinson, 2013	Low risk	Some concern	Low risk	Some concern	Some concern	Some concern
Salai, 2000	High risk	High risk	Some concern	Some concern	Some concern	High risk
Sanders, 2012	Low risk	Low risk	Low risk	Some concern	Some concern	Some concern
Shen, 2001	Some concern	High risk	Some concern	Some concern	Some concern	High risk
Siebenga, 2006	Some concern	Low risk	Low risk	Some concern	Some concern	Some concern
Sletten, 2015	Low risk	Low risk	Low risk	Some concern	Low risk	Some concern
Smekal, 2009	Low risk	Low risk	Low risk	Some concern	Some concern	Some concern
Tamaoki, 2017	Low risk	Low risk	Some concern	Some concern	Some concern	Some concern
Thordarson, 1996	Low risk	Low risk	Some concern	Some concern	Some concern	Some concern
Vinnars, 2008	Low risk	Low risk	Low risk	Some concern	Some concern	Some concern
Virtanen, 2012	Low risk	Low risk	Some concern	Some concern	Some concern	Some concern
Willet, 2016	Low risk	Low risk	Low risk	Some concern	Low risk	Some concern
Woltz, 2017	Low risk	Some concern	Some concern	Some concern	Low risk	Some concern
Wong, 2010	Low risk	Low risk	Some concern	Some concern	Some concern	Some concern
Wood, 2003	Low risk	Low risk	Some concern	Some concern	Some concern	Some concern
Wu, 2018	Low risk	Low risk	Low risk	Some concern	Some concern	Some concern
Zyto, 1997	Low risk	Low risk	Some concern	Some concern	Some concern	Some concern

Study quality was assessed for risk of bias using the Risk of Bias 2.0 tool from the Cochrane Collaboration on trials with results on patient-reported pain, physical function, and/or quality of life [14]. If four or five of the individual domains was found to be associated with some concerns of risk of bias, or if one of them was associated with high risk of bias, the overall risk of bias was rated as high risk

Overall, no trials were judged as low risk of bias and 17 out of 52 trials [18, 21, 22, 27, 28, 31, 49, 52, 56, 58, 60, 61, 67, 72, 75, 77, 79] were associated with a high risk of bias, mainly due to the lack of possibility to blind patients and treatment providers, and lack of pre-registration of the trial in a public trial registry before enrolment of the first patient.

### Harms

#### Synthesis of results

The syntheses of the results are presented in Fig. 5 (SAEs), and in Additional file S2 (deaths and the full forest plot for SAEs).

For 6 out of the 8 fracture types with available data on SAEs from at least two trials, no differences were demonstrated between surgical and non-surgical treatment. For displaced midshaft clavicula fractures (14 trial (*n* = 1394)) [24–37], surgery was associated with a smaller risk of SAEs than non-surgical treatment (RR 0.62 (0.42 to 0.92)). For distal radius fracture (5 trials (n = 436)) [59–61, 65, 80], surgery was associated with a greater risk of SAEs than non-surgical treatment (RR 3.10, 95% CI 1.42 to 6.77).

One trial on unstable malleolar fractures (*n* = 592) [50] and one trial on humeral shaft fractures (*n* = 96) [42] demonstrated fewer SAEs in the surgical compared to the non-surgical group.

There were no differences between surgical and non-surgical treatment in the risk of death for any of the fracture types.

#### Risk of bias

Additional file S3 presents the risk of bias assessment for the individual trials.

Overall, the risk of bias associated with the assessment and reporting of SAEs and death was moderate to high. Only two trials [20, 53] had a score greater than 9 indicating a low risk of bias.

## Discussion

We found a difference in function in favor of surgery (moderate effect) for displaced intraarticular calcaneal fractures (however with large heterogeneity due to a small (*n* = 30), old study) and distal radial fractures (small effect), however, with increased risk of SAEs after surgery for radial fractures. No difference in effect was demonstrated for displaced midshaft clavicular fractures and proximal humeral fractures, scaphoid waist, and thoracolumbar traumatic compression fractures, while surgery for clavicular fractures was associated with reduced risk of SAE. Insufficient data existed for all other fracture types.

The large inconsistency and often missing reporting of SAEs and death in the included trials represent a limitation of our study. The lack of consensus in terms and definitions of complications after treatment of fractures calls for the development and validation of a core set of complications [81]. Another potential limitation of this study relates to our selection of outcomes, as 39 trials were excluded due to insufficient data. Some of the trials had selected composite scores of, e.g., pain and function or other outcomes like time to healing of the fracture, while others did not report data that could be included in meta-analyses, e.g., by reporting pain evaluated on a 5-point Likert scale. For feasibility reasons, we excluded trials that were not in languages understood by any of the authors, which could be a potential bias. However, as only two trials were excluded based on this criterion, the expected impact on the results is considered minimal. Finally, from a clinical point of view, it is common to decide on whether to recommend surgery or not based not only on the fracture type, but also on patient characteristics such as age, work status, and symptom severity. In pragmatic trials, patients are more commonly included without accounting for patient characteristics, which thereby can potentially affect the generalizability of the results from the individual meta-analyses of this study [63].

Although our results could indicate that non-surgical treatment is as effective as surgical treatment for several traumatic fractures in adults, including displaced midshaft clavicular, proximal humeral, scaphoid waist, and thoracolumbar traumatic compression fractures, serious caveats relating to the number of patients studied, heterogeneity and study methodology question the confidence in such a suggestion. First, only 7/19 fracture types had been scrutinized in at least 2 trials with at least 100 patients totally. Second, few and underpowered studies for some fracture types might be part of the explanation for our findings [82], as a previous study found a mean overall study power (1-beta) among 117 trials of traumatic skeletal fractures of 25% [83]. Third, none of the included trials were associated with a low risk of bias for benefits, and only 2/44 (5%) trials were associated with a low risk of bias for SAEs, confirming a previous study summarizing orthopedic trials [82]. In fact, 17/52 (33%) of the trials with data on benefits were associated with a high risk of bias. Finally, the studied fracture types only represent selected types of fractures in selected types of patients. For some fractures (e.g., clavicular and stable lateral malleolar fractures), the natural history of healing without surgical treatment has a good prognosis [84–86]. However, in older persons with lower expectations of function with, e.g., a distal radius or malleolar fracture and more osteoporotic bone, the expected beneficial effect from surgical treatment is typically less than in younger more physically active patients. Thus, some of the studies included represent fracture types suspected to have limited benefits in terms of pain, function, and quality of life from surgical treatment. Other fracture types more obviously in need of surgery (displaced lower arm or hip fractures) is less likely to be subjected to randomization to non-surgical treatment; often termed parachute trials [87]. Despite the mentioned limitations of the SAE reporting, some interesting findings are worth mentioning as our study presents the first overview of SAEs across RCTs of different fractures. While the risk of SAEs was lower from surgical treatment in displaced midshaft clavicular fracture, it was higher in distal radius fractures, and no difference was present for the other six comparisons with the estimated relative risk of SAEs distributed relatively even on both sides of the “no difference in risk” line, dependent on the fracture type. Importantly, most of the findings were based on 2-3 studies, including few patients, precluding any firm conclusions. However, our results do suggest that for some of the more often studied fracture types, like displaced midshaft clavicular fractures, distal radius fractures in older patients, proximal humerus fractures, and traumatic thoraco-lumbar compression fractures, non-surgical treatment might serve as an equally effective and safe treatment as surgical treatment.

Only 20% of the most commonly performed orthopedic procedures, including surgery for fractures, are supported by at least one low risk of bias trial [88]. A search of trials of surgical and non-surgical treatment of fractures in the WHO International Clinical Trials Registry Platform [89] indicates that several ongoing trials will provide data to help build the evidence base for optimal treatment of fractures. Our study is a call to action for more low-risk-of-bias trials powered to detect any difference in benefits and harms between surgical and non-surgical treatment of the most common traumatic skeletal fractures in adults. Although such studies are known to be challenging [90], they are crucial to improve the clinical care of the patients.

## Conclusion

Of 12 fracture types with data from more than one trial, only two demonstrated a difference in function in favor of surgery (moderate effect for displaced intraarticular calcaneal fractures, although affected by a large heterogeneity, and small effect for distal radial fractures), but with greater risk of harms after surgery for radial fractures. We found no difference in effect for displaced midshaft clavicular fractures, proximal humeral fractures, scaphoid waist, and thoracolumbar traumatic compression fractures, while surgery for clavicular fractures was associated with a reduced risk of SAE. Our results also highlight the current paucity of high-quality randomized trials for other common fracture types and a considerable heterogeneity for some of the estimates and risk of bias in a large proportion of available trials.

## Supplementary information

**Additional file 1: S1.** Search strategy for Medline. S2. Assessment of quality of harms assessment and reporting of included trials of surgical and non-surgical treatment of fractures. S3. Full forest plots for all comparisons, including deaths.

## Data Availability

The datasets used and/or analyzed during the current study are available from the corresponding author on reasonable request.

## Abbreviations

SAE: Serious adverse event
BMI: Body mass index
SD: Standard deviation
SMD: Standardized mean difference
RR: Relative risk
