# Supplementary material for: Surgical or non-surgical treatment of traumatic skeletal fractures in adults: systematic review and meta-analysis of benefits and harms
Source: Syst Rev. 2020 Aug 13;9:179. doi: 10.1186/s13643-020-01424-4 (PMC7425058; doi:10.1186/s13643-020-01424-4)
Supplement: Supplementary file 1 — Additional file 1: S1. Search strategy for Medline. S2. Assessment of quality of harms assessment and reporting of included trials of surgical and non-surgical treatment of fractures. S3. Full forest plots for all comparisons, including deaths. [file 13643_2020_1424_MOESM1_ESM.docx]

**SUPPLEMENTAL FILES**

S1. Search strategy for Medline

(randomi*[TIAB] OR "randomized controlled trial"[Publication Type] OR "randomized controlled trials as topic"[MeSH Terms])

AND

(“Joints”[MeSH] OR “Joint*”[TIAB] OR "Fractures, Bone"[Mesh] OR fracture*[TIAB] OR "Ligaments, Articular"[MeSH] OR (“ligament*”[TIAB] AND “articular”[TIAB]) OR “Anterior Cruciate Ligament”[MeSH] OR “Anterior Cruciate Ligament”[TIAB] OR “Achilles Tendon”[MeSH] OR “Achilles Tendon”[TIAB] OR “Rotator Cuff”[MeSH] OR “Rotator Cuff”[TIAB] OR "Spine"[Mesh] OR Spina*[TIAB])

AND

(“general surgery”[MeSH] OR operativ*[TIAB] OR “surgical procedures, operative"[MeSH] OR surger*[TIAB] OR surgical[TIAB] OR arthroscopy*[TIAB] OR arthroscopic[TIAB] OR reconstruction*[TIAB] OR “surgery”[Subheading] OR “reconstructive surgical procedures”[MeSH] OR reconstructive[TIAB] OR “reconstructive surgical procedures”[TIAB] OR reconstruction*[TIAB] OR meniscectom*[TIAB] OR debridement[TIAB])

AND

(Nonsurgical*[TIAB] OR nonoperativ*[TIAB] OR exercis*[TIAB] OR Exercise therapy[MeSH] OR “Resistance training”[MeSH] OR Resistance training[TIAB] OR Strength Training[TIAB] OR placebos[MeSH] OR (sham[TIAB] AND surger*[TIAB]) OR delayed*[TIAB] OR placebo*[TIAB] OR control*[TIAB] OR usual care[TIAB])

S2. Full forest plots for all comparisons, including deaths

Forest plot of effects of surgical and non-surgical treatment on pain. Fracture sites are in alphabetic order to increase overview.


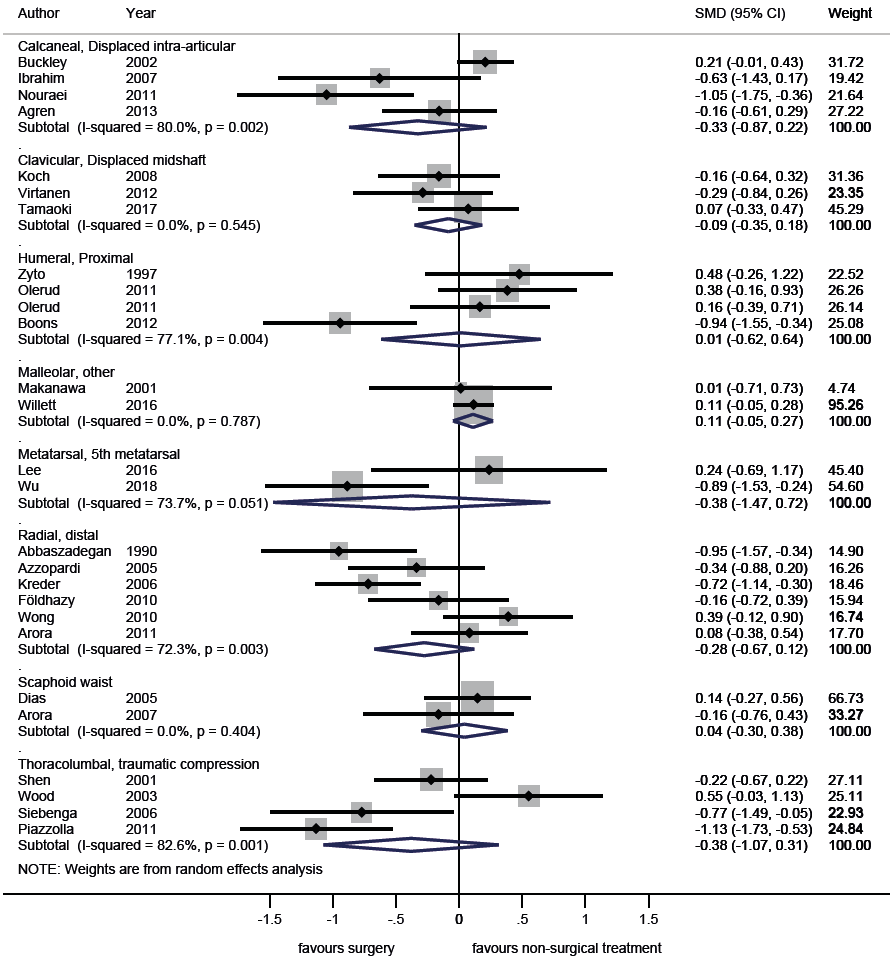


Forest plot of effects of surgical and non-surgical treatment on function. Fracture sites are in alphabetic order.


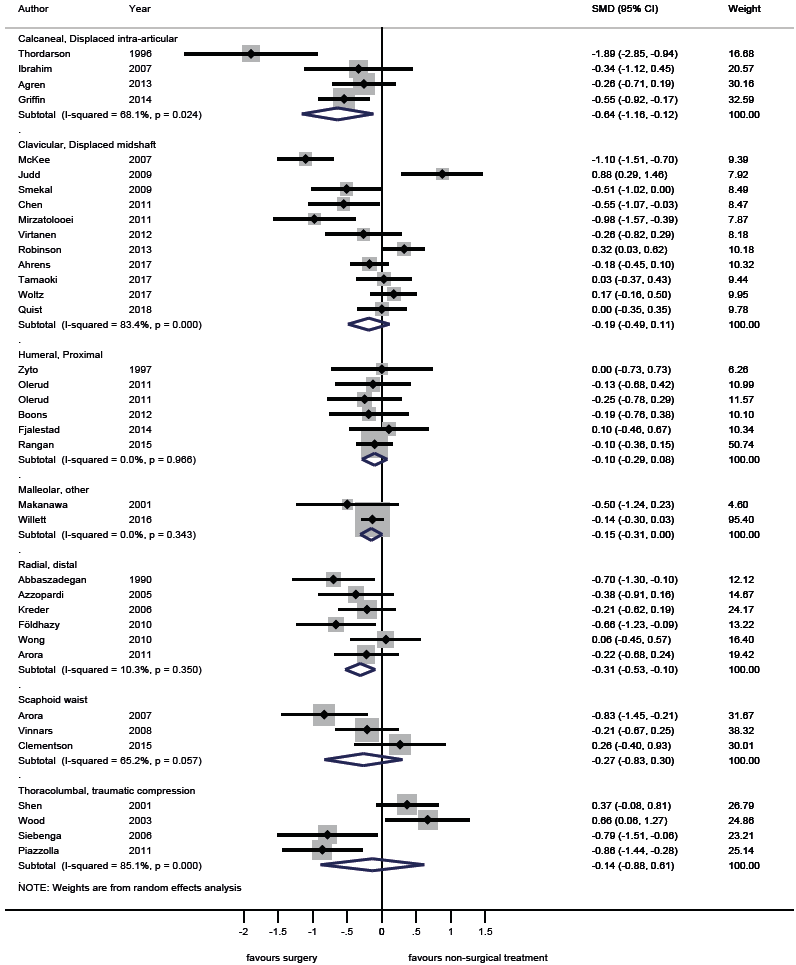


Forest plot of effects of surgical and non-surgical treatment on quality of life. Fracture sites are in alphabetic order.


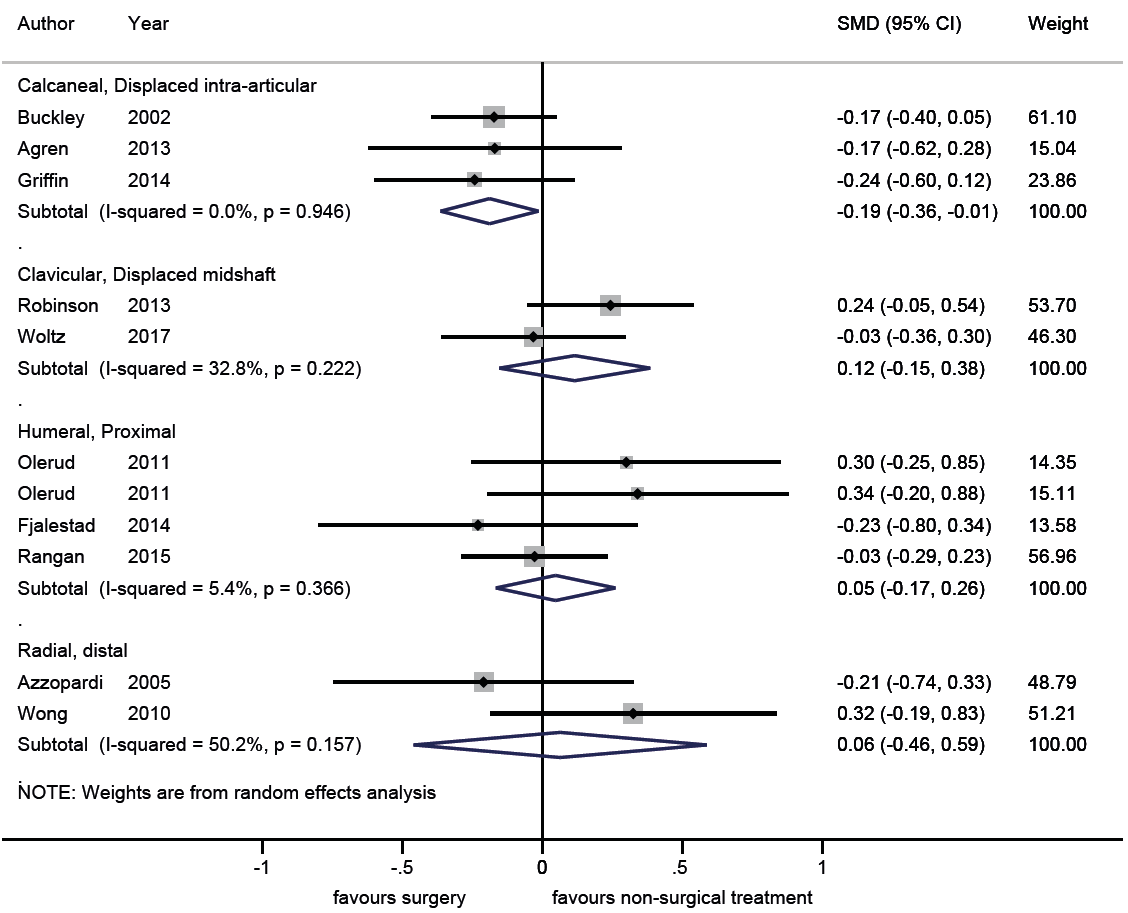


Forest plot of effects of surgical and non-surgical treatment on serious adverse events. Fracture sites are in alphabetic order.


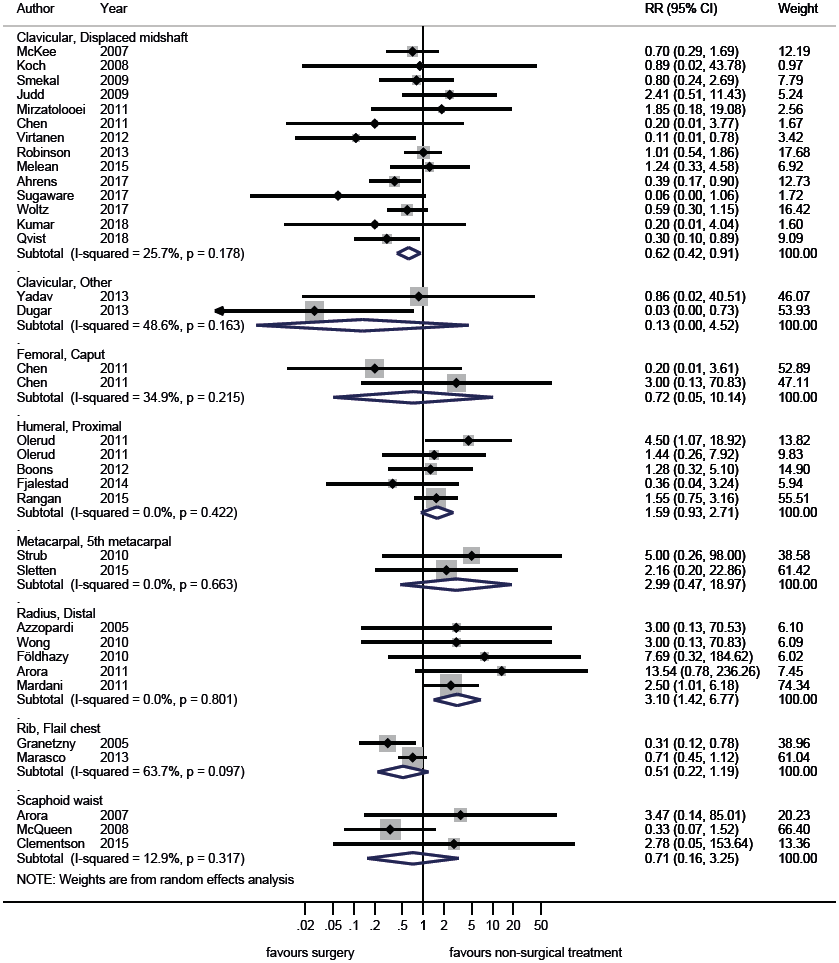


Forest plot of effects of surgical and non-surgical treatment on death. Fracture sites are in alphabetic order.


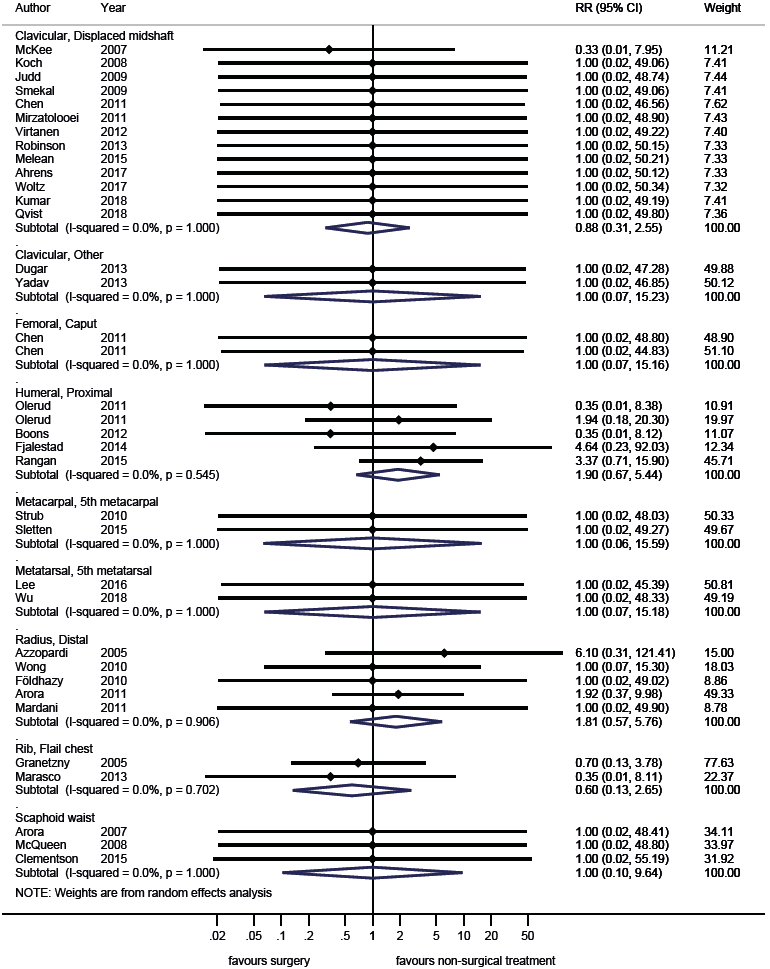


S3. Assessment of quality of harms assessment and reporting of included trials of surgical and non-surgical treatment of fractures

| **Author, Year** | **1** | **2** | **3** | **4** | **5** | **6** | **7** | **8** | **9** | **10** | **11** | **12** | **13** | **14** | **15** | **TOTAL** |
| --- | --- | --- | --- | --- | --- | --- | --- | --- | --- | --- | --- | --- | --- | --- | --- | --- |
| Ahrens, 2017 | 1 | 0 | 0 | 0 | 0 | 0 | 0 | N/A | 0 | 0 | 1 | 1 | 0 | 1 | 0 | 4 |
| Arora, 2007 | 0 | 0 | 0 | 1 | 0 | 0 | 0 | N/A | 0 | 0 | 1 | 1 | 0 | 1 | 0 | 4 |
| Arora, 2011 | 1 | 0 | 0 | 1 | 0 | 0 | 0 | N/A | 0 | 0 | 1 | 1 | 1 | 1 | 1 | 7 |
| Azzopardi, 2005 | 0 | 0 | 0 | 1 | 0 | 0 | 0 | N/A | 0 | 0 | 1 | U | 1 | 1 | 0 | 4 |
| Boons, 2012 | 0 | 0 | 0 | 1 | 1 | 0 | 1 | 1 | 1 | 0 | 1 | 1 | 0 | 1 | 0 | 8 |
| Chen, 2011 | 0 | 0 | 0 | 1 | 0 | 0 | 0 | N/A | 0 | 0 | U | 1 | 1 | 1 | 0 | 4 |
| Chen, 2011 | 0 | 0 | 0 | 1 | 1 | 0 | 0 | N/A | 0 | 0 | U | 1 | 1 | 1 | 1 | 6 |
| Chen, 2011 | 1 | 0 | 0 | 1 | 0 | 0 | 0 | N/A | 0 | 0 | 1 | 1 | 1 | 1 | 1 | 7 |
| Clementson, 2015 | 0 | 0 | 0 | 1 | 0 | 0 | 0 | N/A | 0 | 0 | 1 | 1 | 1 | 1 | 0 | 5 |
| Duckworth, 2017 | 0 | 0 | 0 | 1 | 0 | 0 | 0 | N/A | 1 | 0 | 1 | 1 | 1 | 1 | 1 | 7 |
| Dugar, 2013 | 0 | 0 | 0 | 0 | 0 | 0 | 0 | N/A | 0 | 0 | 0 | 0 | 0 | 1 | 0 | 1 |
| Fjalestad, 2014 | 0 | 0 | 0 | 1 | 0 | 0 | 0 | N/A | 0 | 0 | 1 | 1 | 1 | 1 | 0 | 5 |
| Földhazy, 2010 | 0 | 0 | 0 | 1 | 1 | 0 | 0 | N/A | 1 | 0 | 1 | 1 | 1 | 1 | 0 | 7 |
| Granetzny, 2005 | 0 | 0 | 0 | 1 | 0 | 0 | 0 | N/A | 0 | 0 | 1 | 0 | 1 | 1 | 1 | 5 |
| Griffin, 2014 | 1 | 0 | 0 | 1 | 1 | 0 | 1 | 1 | 1 | 1 | 1 | 1 | 1 | 1 | 1 | 12 |
| Hussain, 2017 | 0 | 0 | 0 | 0 | 0 | 0 | 0 | N/A | 0 | 0 | 0 | 1 | 0 | 1 | 1 | 3 |
| Judd, 2009 | 0 | 0 | 0 | 1 | 1 | 0 | 0 | N/A | 1 | 0 | 1 | 1 | 1 | 1 | 0 | 7 |
| Koch, 2008 | 0 | 0 | 0 | 0 | 0 | 0 | 0 | N/A | 0 | 0 | 0 | 0 | 1 | 1 | 1 | 3 |
| Kumar, 2018 | 0 | 0 | 0 | 0 | 0 | 0 | 0 | N/A | 0 | 0 | 0 | 0 | 1 | 1 | 0 | 2 |
| Marasco, 2013 | 0 | 1 | 0 | 1 | 0 | 0 | 0 | N/A | 1 | 0 | 1 | 1 | 1 | 1 | 1 | 8 |
| Mardani, 2011 | 0 | 0 | 0 | 1 | 1 | 0 | 0 | N/A | 1 | 0 | 0 | 0 | 1 | 1 | 0 | 5 |
| Matsunaga, 2017 | 0 | 0 | 1 | 1 | 1 | 0 | 0 | N/A | 1 | 0 | 1 | 1 | U | 1 | 1 | 8 |
| McKee, 2007 | 1 | 0 | 0 | 1 | 0 | 0 | 0 | N/A | 0 | 0 | 1 | 1 | 0 | 1 | 1 | 6 |
| McQueen, 2008 | 1 | 0 | 0 | 0 | 0 | 0 | 0 | N/A | 1 | 0 | 1 | 0 | 0 | 1 | 0 | 4 |
| Melean, 2015 | 0 | 0 | 0 | 0 | 0 | 0 | 0 | N/A | 0 | 1 | 1 | 0 | 0 | 0 | 1 | 3 |
| Mirzatolooei, 2011 | 0 | 0 | 0 | 0 | 1 | 0 | 0 | N/A | 1 | 0 | 0 | 1 | 1 | 1 | 1 | 6 |
| Mittal, 2017 | 1 | 1 | 0 | 1 | 1 | 0 | 0 | N/A | 1 | 0 | 1 | 1 | 0 | 1 | 1 | 9 |
| Olerud, 2011 | 0 | 0 | 0 | 1 | 0 | 0 | 0 | N/A | 1 | 0 | 0 | 1 | 1 | 1 | 0 | 5 |
| Olerud, 2011 | 0 | 0 | 0 | 1 | 0 | 0 | 0 | N/A | 1 | 0 | 0 | 1 | 1 | 1 | 0 | 5 |
| Piazzolla, 2011 | 0 | 0 | 0 | 1 | 0 | 0 | 0 | N/A | 0 | 0 | U | 1 | 1 | 1 | 0 | 4 |
| Qvist, 2018 | 0 | 0 | 0 | 0 | 0 | 0 | 0 | N/A | 1 | 0 | 0 | 1 | 0 | 1 | 1 | 4 |
| Rangan, 2015 | 1 | 0 | 0 | 1 | 1 | 0 | 0 | N/A | 1 | 0 | 1 | 1 | 1 | 1 | 1 | 9 |
| Robinson, 2013 | 0 | 0 | 0 | 1 | 1 | 0 | 1 | 1 | 1 | 0 | 1 | 1 | 0 | 1 | 0 | 8 |
| Sanders, 2012 | 0 | 0 | 0 | 1 | 1 | 0 | 1 | 1 | 1 | 0 | 1 | 1 | 1 | 1 | 1 | 10 |
| Sletten, 2015 | 1 | 0 | 0 | 0 | 0 | 0 | 0 | N/A | 1 | 0 | 1 | 1 | 1 | 1 | 1 | 7 |
| Smekal, 2009 | 0 | 0 | 0 | 1 | 0 | 0 | 0 | N/A | 0 | 0 | 1 | 1 | 0 | 1 | 1 | 5 |
| Strub, 2010 | 0 | 0 | 0 | 0 | 0 | 0 | 0 | N/A | 1 | 0 | 0 | 1 | 1 | 0 | 0 | 3 |
| Tamaoki, 2017 | 0 | 0 | 1 | 0 | 0 | 0 | 0 | N/A | 1 | 0 | 1 | 1 | 1 | 1 | 1 | 7 |
| Virtanen, 2012 | 0 | 0 | 0 | 1 | 0 | 0 | 0 | N/A | 0 | 0 | 1 | 1 | 0 | 1 | 1 | 5 |
| Willet, 2016 | 1 | 1 | 0 | 1 | 0 | 0 | 0 | N/A | 1 | 0 | 1 | 1 | 1 | 1 | 1 | 9 |
| Woltz, 2017 | 0 | 0 | 0 | 0 | 0 | 0 | 1 | 0 | 1 | 0 | 1 | 1 | 0 | 1 | 1 | 6 |
| Wong, 2010 | 0 | 0 | 0 | 1 | 0 | 0 | 0 | N/A | 0 | 0 | 0 | 1 | 1 | 1 | 0 | 4 |
| Wu, 2018 | 0 | 0 | 0 | 0 | 0 | 0 | 0 | N/A | 1 | 0 | 0 | 1 | 1 | 1 | 0 | 4 |
| Yadav, 2015 | 0 | 0 | 0 | 0 | 0 | 0 | 1 | 1 | 1 | 0 | 0 | 1 | 1 | 1 | 0 | 6 |

For serious adverse events (SAEs) study quality was assessed independently on trials with results on SAEs using the 15-point McMaster tool for assessing quality of harms assessment and reporting in study reports (McHarm);^15^  0/1:No/Yes; 1. Were the harms PREDEFINED using standardized or precise definitions?; 2. Were SERIOUS events precisely defined?; 3. Were SEVERE events precisely defined?; 4. Were the number of DEATHS in each study group specified OR were the reason(s) for not specifying them given?; 5. Was the mode of harms collection specified as ACTIVE?; 6. Was the mode of harms collection specified as PASSIVE?; 7. Did the study specify WHO collected the harms?; 8. Did the study specify the TRAINING or BACKGROUND of who ascertained the harms?; 9. Did the study specify the TIMING and FREQUENCY of collection of the harms?; 10. Did the author(s) use STANDARD scale(s) or checklist(s) for harms collection?; 11. Did the authors specify if the harms reported encompass ALL the events collected or a selected SAMPLE?; 12. Was the NUMBER of participants that withdrew or were lost to follow-up specified for each study group?; 13. Was the TOTAL NUMBER of participants affected by harms specified for each study arm?; 14. Did the author(s) specify the NUMBER for each TYPE of harmful event for each study group?; 15. Did the author(s) specify the type of analyses undertaken for harms data?; N/A: Given in item 8, if the study had not indicated who collected the harms (item 7); U: Unclear, whether the specific item was fulfilled or not.
